# Roxadustat: More Than an Erythropoietic Agent?

**DOI:** 10.1016/j.ekir.2024.06.042

**Published:** 2024-10-26

**Authors:** Andrea Angeletti, Paolo Cravedi

**Affiliations:** 1 Division of Nephrology, Dialysis, Transplantation, IRCCS Istituto Giannina Gaslini, Genova, Italy; 2 Translational Transplant Research Center and Department of Medicine, Icahn School of Medicine at Mount Sinai, New York, USA

To the Editor:

We congratulate Kong *et al.*^1^ for their randomized study showing that the oral hypoxia-inducible factor prolyl hydroxylase (HIF-PH) inhibitor, roxadustat increases hemoglobin in kidney transplant recipients with posttransplant anemia. Despite proven efficacy, the widespread use of HIF-PHs will largely depend on future studies clearly characterizing the safety profile of these molecules in various populations of patients with kidney failure. A recent meta-analysis showed that the cardiovascular risk of HIF-PHs is similar to that of other erythropoiesis-stimulating agents^2^; however, concerns about the potential risks of infections and tumors persist, especially in light of the evidence that HIF can directly promote tumor cell growth.

HIF-PHs promote erythropoiesis by stabilizing the transcription factor HIF-α that increases endogenous production of erythropoietin (EPO). Given that immune cells produce EPO, we contend that at least part of the nonerythropoietic effects associated with HIF-PHs are simply due to increased EPO production. We showed that EPO directly inhibits effector T cells and promotes induction of regulatory T cells.^3^ These immune modulatory effects can concur to the increased susceptibility to cancer and infections of patients on prolonged HIF-PH treatment.S1^,^S2 In addition, immune modulating effects are responsible for the prolonged graft survival of EPO-treated miceS3 and possibly, humans.^4^

Intriguingly, Kong *et al.*^1^ showed that, despite similar physical activity, patients on roxadustat have a significant decrease in total cholesterol, triglycerides, low-density lipoprotein cholesterol, and high-density lipoprotein cholesterol than controls. These affects could also be attributed to the EPO signaling in the peripheral adipose tissue.^5^

Theoretically, promoting endogenous EPO production might have a different safety or efficacy profile than exogenous EPO administration. While more evidence deciphering differences between HIF-PHs and other erythropoiesis-stimulating agents is generated, we suggest that future studies monitor circulating EPO levels to test the hypothesis that they are the primary determinants of nonerythropoietic effects of HIF-PHs.

## Disclosure

All the authors declared no competing interests.

## Supplementary Material

Supplementary File (PDF) **Supplementary References**.

## Footnotes

Supplementary File (PDF)
**Supplementary References**.

## References

[1] Kong W., Wu X., Shen Z. (2024). The efficacy and safety of roxadustat for the treatment of posttransplantation anemia: a randomized study. Kidney Int Rep.

[2] Jhee J.H., Oh D., Seo J. (2023). Short-term blood pressure variability and incident CKD in patients with hypertension: findings from the cardiovascular and metabolic disease etiology research center-high risk (CMERC-HI) study. Am J Kidney Dis.

[3] Donadei C., Angeletti A., Cantarelli C. (2019). Erythropoietin inhibits SGK1-dependent TH17 induction and TH17-dependent kidney disease. JCI Insight.

[4] Guglielmo C., Bin S., Cantarelli C. (2021). Erythropoietin reduces auto- and alloantibodies by inhibiting T follicular helper cell differentiation. J Am Soc Nephrol.

[5] Li J., Yang M., Yu Z., Tian J., Du S., Ding H. (2019). Kidney-secreted erythropoietin lowers lipidemia via activating JAK2-STAT5 signaling in adipose tissue. EBiomedicine.

