# Supplementary material for: Roxadustat: More Than an Erythropoietic Agent?
Source: Kidney Int Rep. 2024 Oct 26;10(1):275. doi: 10.1016/j.ekir.2024.06.042 (PMC11725816; doi:10.1016/j.ekir.2024.06.042)
Supplement: Supplementary File (PDF) — Supplementary References. [file mmc1.pdf]

Supplementary Material

Roxadustat: more than an erythropoietic agent?

Andrea Angeletti[1], Paolo Carvedi[2]

[1]Division of Nephrology, Dialysis, Transplantation, IRCCS Istituto Giannina Gaslini, Genova, Italy;
[2]Translational Transplant Research Center (TTRC) and Department of Medicine, Icahn School of Medicine at Mount Sinai, New York, NY, USA;

Table of Contents

Supplementary References ***Pag.3***

Supplementary References

S1. Haase VH. Hypoxia-inducible factor–prolyl hydroxylase inhibitors in the treatment of anemia of chronic kidney disease. *Kidney Int Suppl*. 2021; 11, 8–25. doi: 10.1016/j.kisu.2020.12.002

S2. Yap DYH, McMahon LP, Hao CM et al. Recommendations by the Asian Pacific society of nephrology (APSN) on the appropriate use of HIF-PH inhibitors. *Nephrology (Carlton)*. 2021;26,105-118. doi: 10.1111/nep.13835.

S3. Horwitz JK, Bin S, Fairchild RL et al. Linking erythropoietin to Treg-dependent allograft survival through myeloid cells. *JCI Insight*. 2022;23,7(10):e158856. doi: 10.1172/jci.insight.158856.
